# Pro‐apoptotic Noxa is involved in ablative focal irradiation‐induced lung injury

**DOI:** 10.1111/jcmm.13014

**Published:** 2016-11-15

**Authors:** Jee‐Youn Kim, Yong‐Min An, Won Hoon Choi, Jin‐Mo Kim, Samju Cho, Byung Rok Yoo, Jeong Wook Kang, Yun‐Sil Lee, Yoon‐Jin Lee, Jaeho Cho

**Affiliations:** ^1^ Department of Radiation OncologyYonsei University College of MedicineSeoulSouth Korea; ^2^ College of Pharmacy and Division of Life and Pharmaceutical ScienceEwha Womans UniversitySeoulSouth Korea; ^3^ Division of Radiation EffectsKorea Institute of Radiological and Medical SciencesSeoulSouth Korea

**Keywords:** Noxa, radiation, lung injury, apoptosis

## Abstract

Although lung injury including fibrosis is a well‐documented side effect of lung irradiation, the mechanisms underlying its pathology are poorly understood. X‐rays are known to cause apoptosis in the alveolar epithelial cells of irradiated lungs, which results in fibrosis due to the proliferation and differentiation of fibroblasts and the deposition of collagen. Apoptosis and BH3‐only pro‐apoptotic proteins have been implicated in the pathogenesis of pulmonary fibrosis. Recently, we have established a clinically analogous experimental model that reflects focal high‐dose irradiation of the ipsilateral lung. The goal of this study was to elucidate the mechanism underlying radiation‐induced lung injury based on this model. A radiation dose of 90 Gy was focally delivered to the left lung of C57BL/6 mice for 14 days. About 9 days after irradiation, the mice began to show increased levels of the pro‐apoptotic protein Noxa in the irradiated lung alongside increased apoptosis and fibrosis. Suppression of Noxa expression by small interfering RNA protected cells from radiation‐induced cell death and decreased expression of fibrogenic markers. Furthermore, we showed that reactive oxygen species participate in Noxa‐mediated, radiation‐induced cell death. Taken together, our results show that Noxa is involved in X‐ray‐induced lung injury.

## Introduction

Stereotactic body radiotherapy (SBRT) is an advanced technology used to deliver an ablative dose in a single fraction or few fractions to deep‐seated tumours. It results in dramatically higher rates of local tumour control compared with conventional fractionated radiotherapy (CFRT), especially for smaller tumours located in the periphery of large organs such as the liver and lungs 1, 2. Thus, SBRT has emerged as a standard treatment for high‐risk patients with early‐stage non‐small cell lung cancer 3. In our previous studies 4, we established an experimental model and an image‐guided animal irradiation system for the study of ablative dose per fraction irradiation similar to that used with SBRT at volumes analogous to those used in human beings. Ablative irradiation can induce fibrosis in the area of the lung irradiation. Fibrosis is an important cause of lung diseases including asthma, interstitial lung diseases and idiopathic pulmonary fibrosis (IPF) 5. In these diseases, fibrosis often coexists with lung epithelial cell apoptosis 6, and blocking of apoptosis has resulted in attenuated fibrotic responses in various experimental systems 7, 8. However, the mechanisms underlying the fibrotic and apoptotic responses in these lung disorders are not fully understood. In this study, we investigated the molecular mechanism underlying focal irradiation‐induced lung fibrosis by taking advantage of this image‐guided animal irradiation system.

The respiratory epithelium plays a critical role in lung homoeostasis 9. Severe injury, delayed repair or extensive loss of the alveolar epithelium stimulates macrophage influx and pro‐inflammatory cytokine elaboration. This results in fibrosis caused by the proliferation and differentiation of fibroblasts and the deposition of collagen 9, 10. Although extensive evidence from several different animal models supports the importance of alveolar epithelium apoptosis in the pathogenesis of pulmonary fibrosis 11, 12, the molecular mechanisms of apoptosis following X‐ray irradiation and their downstream effector molecules are poorly understood.

We used microarrays to identify differentially expressed genes that respond to X‐ray irradiation; we found Noxa, which is a pro‐apoptotic member of the BH3‐only Bcl‐2 family of proteins and is up‐regulated during p53‐mediated apoptosis 13. Here, we report that Noxa is transcriptionally activated during X‐ray irradiation and it mediates X‐ray‐induced alveolar epithelial cell (AEC) death. In addition, we show that Noxa‐mediated cell death was resulted by generation of reactive oxygen species (ROS). Inhibition of endogenous Noxa expression by siRNA suppressed X‐ray‐induced cell death and reduced the expression of fibrogenic markers induced by X‐rays. On the basis of our results, we propose that Noxa is involved in X‐ray‐induced lung fibrosis.

## Materials and methods

### Mouse irradiation

Radiation was delivered to a small volume of the left lung using an image‐guided small animal irradiation system coupled with X‐Rad 320 equipment (Precision, North Branford, CT, USA) 14. To mimic clinical SBRT conditions by irradiating only a small volume, we selected a 3‐mm collimator and a dose of 90 Gy. To observe sequential pathological changes, mice were killed at days 0, 5, 9 and 14 after irradiation. Three mice were allocated to each group and the experiment was repeated twice.

### Preparation of lung tissues, immunohistochemistry, and the TUNEL assay

For histological examination, lung tissues were stained sequentially with haematoxylin and eosin, and Masson's trichrome (Sigma‐Aldrich, St. Louis, MO, USA) staining was carried out as previously described 15. Immunohistochemical staining was carried out using anti‐Noxa (Abcam, Cambridge, MA, USA) and anti‐8‐OHdG (Genox, Baltimore, MD, USA) antibodies. A terminal deoxynucleotidyl transferase‐mediated deoxyuridine‐5‐triphosphate‐biotin nick end labelling (TUNEL) assay (ApopTag; Millipore, Billerica, MA, USA) was performed according to the manufacturer's instructions.

### Cell culture

MLE12 and L132 mouse and human lung epithelial cells were grown in DMEM supplemented with 10% foetal bovine serum at 37°C in a humidified 5% (v/v) CO_2_ atmosphere. The cells were seeded at 1.0 × 10^6^ cells/60 mm plate. After 24 hrs, the cells were washed with serum‐free medium and stored prior to the experiments.

### RT‐PCR and Western blotting

Total RNA was isolated from the lungs and cells using the TRIzol reagent from Invitrogen (Carlsbad, CA, USA) according to the manufacturer's instructions. Extracted proteins were separated by SDS‐PAGE, the membranes were probed with primary antibody, followed by incubation with horseradish peroxidase‐coupled secondary antibody. Detection was performed with a chemiluminescence‐based detection kit (Bio‐Rad, Hercules, CA, USA).

### Adenoviral Noxa overexpression system

Pre‐made human Noxa adenovirus (Ad‐Noxa) was purchased from ABM Inc. (Richmond, BC, Canada). To transiently express Noxa, cells were infected with Ad‐Noxa and incubated in serum‐free medium for 12 hrs, before changing to a fresh complete medium.

### Suppression of Noxa expression by siRNA

For siRNA inhibition studies, the cells were transfected with Noxa or control siRNA (Santa Cruz Biotechnology Inc., Santa Cruz, CA, USA) at a final concentration of 100 nM using Lipofectamine 2000 (Invitrogen) according to the manufacturer's instructions. After transfection, the cells were harvested for protein extraction and additional analysis.

### Immunocytochemistry

Cells were cultured on coverslips coated with poly‐l‐lysine, fixed with 4% paraformaldehyde in PBS and permeabilized with 0.1% Triton X‐100. The cells were incubated with the anti‐Noxa or anti‐phospho‐H2AX (S139; Cell Signaling Technology, Danvers, MA, USA) at 4°C overnight, and then stained with a secondary. These cells were viewed by confocal microscopy (LSM 700; Zeiss, Jena, Germany).

### Flow cytometry analysis of apoptosis

Flow cytometry analysis was performed to identify apoptosis by Annexin V‐FITC/Propidium Iodide apoptosis detection kit (Cell Signaling Technology). After treatment, cells (1 × 10^5^ cells/30 mm dish) were harvested and stained with Annexin V‐FITC/PI according to the manufacturer's procedure. The cells were analyzed by FACS Verse flow cytometer (Beckman Coulter, Brea, CA, USA).

### Luciferase assay

The mouse Noxa promoter sequence was amplified by PCR from mouse kidney genomic DNA using forward and reverse primers containing KpnI and HindIII sites respectively. The Noxa promoter fragment was in‐frame cloned into the pGL2 luciferase reporter vector. Luciferase activity was measured in samples containing equivalent amounts of protein using a luminometer and luciferase assay reagents (Promega Corp., Madison, WI, USA).

### WST‐1‐based cytotoxicity assay

The cell proliferation reagent WST‐1 was purchased from Roche (Roche Hungary Ltd., Budapest, Hungary). After treatment, the WST‐1 assays were performed in triplicate according to the manufacturer's instructions. Briefly, cells were supplemented with the WST‐1 assay reagent and incubated for 2 hrs. The plates were then read on a microplate spectrophotometer at 450 nm and the per cent cytotoxicity was calculated.

### Statistics

Statistical analysis of the data was conducted using Prism 5 software (GraphPad Software Inc., San Diego, CA, USA). All values are presented as the mean ± S.E.M. Differences between the means of the control and treatment samples were determined by an unpaired *t*‐test using Welch's correction. Results with a *P* < 0.05 were considered statistically significant.

## Results

### Effect of radiation on gross morphology and histopathological analysis

**Figure 1:**
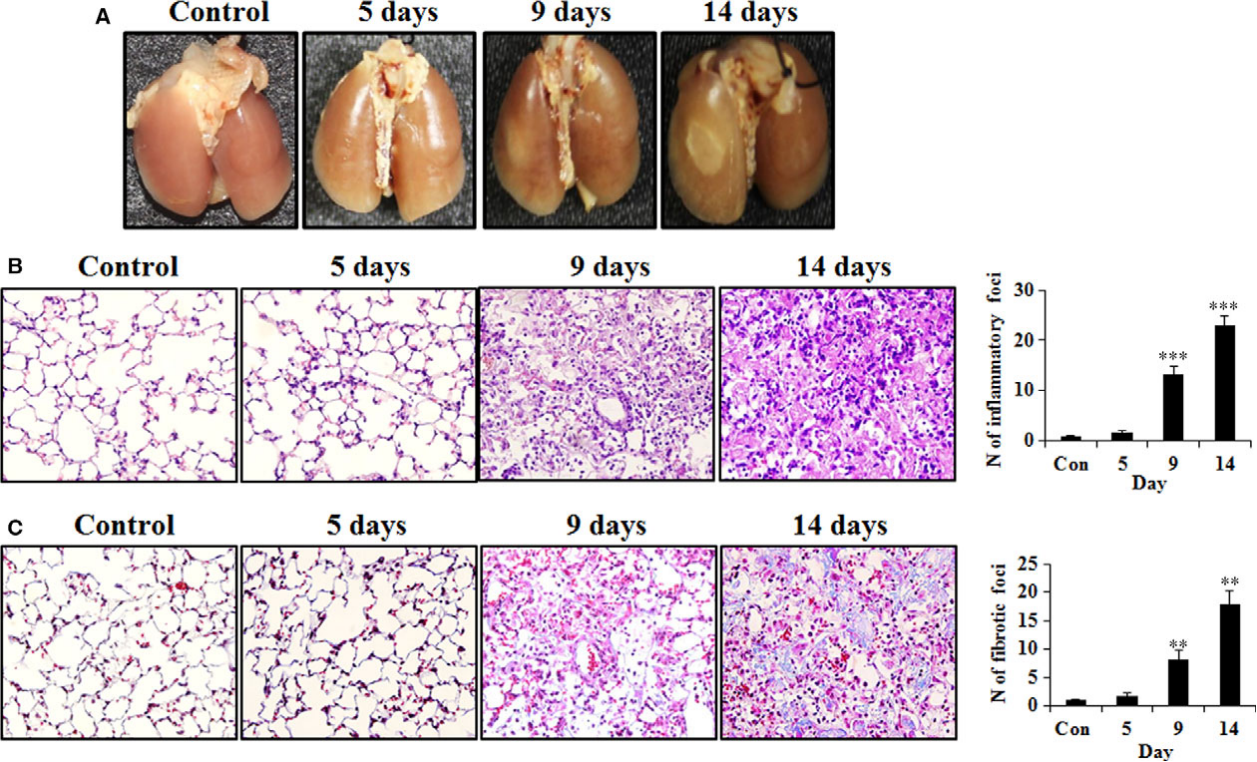
Morphologic observation in control and irradiation (IR) groups. (**A**) Representative gross findings. Mice were killed at the indicated time‐points after irradiation, and the lungs were immersed in fixation solution for several days. Lungs were photographed after complete fixation. Hematoxylin and eosin‐stained (**B**) and Masson's trichrome‐stained (**C**) irradiated lung sections from a minimum of three mice were examined at each time‐point. Representative images of the major findings are shown (magnification: × 400). Quantification of inflammation and fibrosis score is shown in each right panel. **P < 0.01, ***P < 0.001 *versus* control.

To evaluate the effects of radiation on lung surfaces, we observed morphological abnormalities on left lung surfaces compared with the control for irradiation (IR) group at 5, 9, and 14 days after treatment with X‐rays at a dose of 90 Gy. After 9 days, the irradiated area exhibited a white ring‐like appearance (Fig. 1A). The number of inflammatory cells and the formation of intra‐alveolar hyaline membranes gradually increased in the irradiated region (Fig. 1B). To evaluate fibrosis, lung sections were stained with Masson's trichrome. The collagen deposition and number of fibrotic foci were increased in IR group (Fig. 1C).

### Effects of X‐ray irradiation on Noxa expression

**Figure 2:**
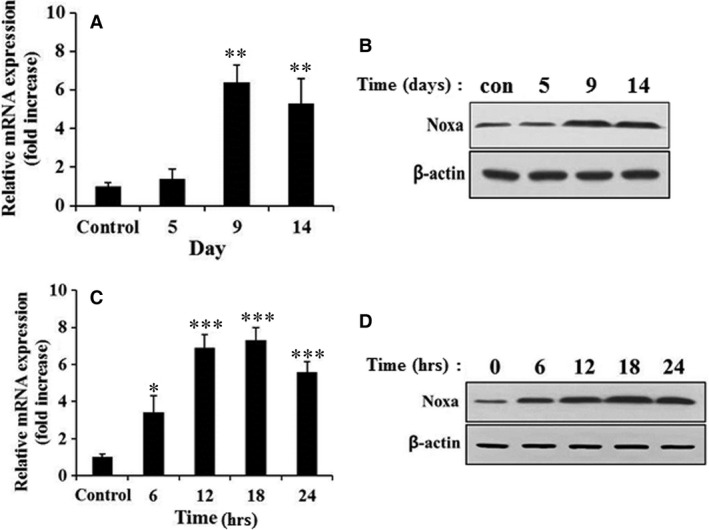
Effect of irradiation on Noxa mRNA and protein expression. Quantitative RT‐PCR analysis (**A** and **C**) and western blotting (**B** and **D**) showed that irradiation increased Noxa expression in the mouse lungs and MLE12 cells. cDNA was synthesized from the total RNAs extracted from irradiated mouse lungs (**A**) and MLE12 cells (**C**) exposed to X‐rays and subjected to RT‐PCR analysis. The Noxa expression level of the control was arbitrarily defined as 1. *P < 0.05, **P < 0.01, ***P < 0.001 *versus* control. Cell lysates (20 μg) extracted from X‐ray‐irradiated mouse lungs (**B**) and MLE‐12 cells (**D**) were subjected to western blotting analysis using polyclonal anti‐Noxa and β‐actin antibodies.

To identify which genes responded to X‐ray irradiation, microarray analysis was performed with irradiated mouse lungs (control, X‐ray dose of 90 Gy for 14 days) (Table S1). Of the genes that were differentially expressed in comparison with the control at 14 days, we focused on *Pmaip1*, the gene that encodes the Noxa protein. Noxa is a pro‐apoptotic member of the BH3‐only Bcl‐2 family. It is known to be a downstream effector of p53‐induced apoptosis through genotoxic stress produced by, for example, DNA damage or X‐rays 13. To confirm the microarray results, RT‐PCR and western blotting were performed with X‐ray‐irradiated mouse lung tissue. As presented in Figure 2A and B, Noxa mRNA and protein levels were significantly increased. To confirm the effect of the X‐rays on *in vitro* Noxa expression in the cell line system, MLE12 mouse lung epithelial cells were treated with X‐ray radiation at a dose of 10 Gy for 0, 6, 12, 18 and 24 hrs, and then RT‐PCR and western blotting were performed. The irradiated cells showed a significant increase in the levels of Noxa mRNA and protein following 6 hrs of IR (Fig. 2C and D).

### Noxa promoter responds to X‐ray irradiation

**Figure 3:**
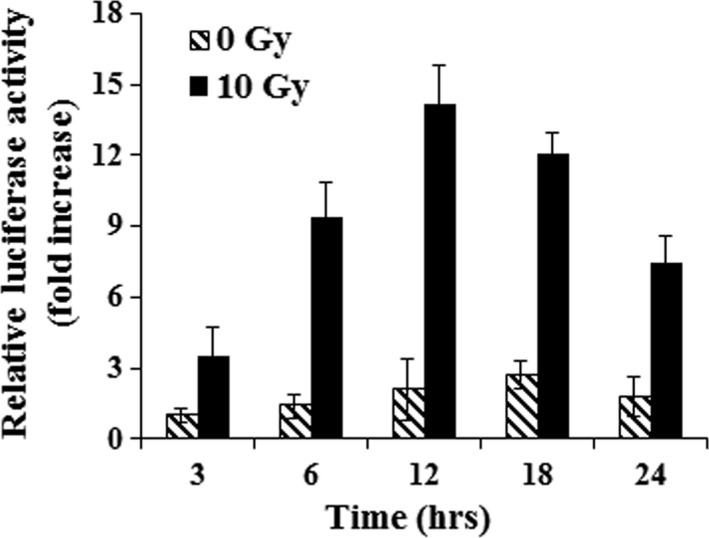
Activation of Noxa promoter by X‐rays. MLE12 cells were transiently transfected with 1 μg luciferase reporter plasmid. After 24 hrs of transfection, the cells were subjected to X‐rays for the indicated time periods and luciferase activity was determined. The means ± S.D. of three independent experiments are shown.

The increase in Noxa expression following exposure to X‐rays (Fig. 2) prompted us to determine whether the promoter of Noxa might respond to X‐rays. A promoter assay was performed with the luciferase reporter gene of Noxa. We transfected MLE12 cells with pGL2‐Noxa for 24 hrs, and the cells were exposed to X‐rays at a dose of 10 Gy for an additional 3, 6, 12, 18 and 24 hrs. An increase in the luciferase activities was initially found at 3 hrs (3.5‐fold) with a time‐dependent increase up to 12 hrs (9.4‐, 14.2‐, 12.1‐ and 7.5‐fold for 6, 12 18, and 24 hrs, respectively), indicating that the Noxa promoter responded to X‐rays (Fig. 3).

### Noxa facilitates X‐ray‐induced apoptosis

**Figure 4:**
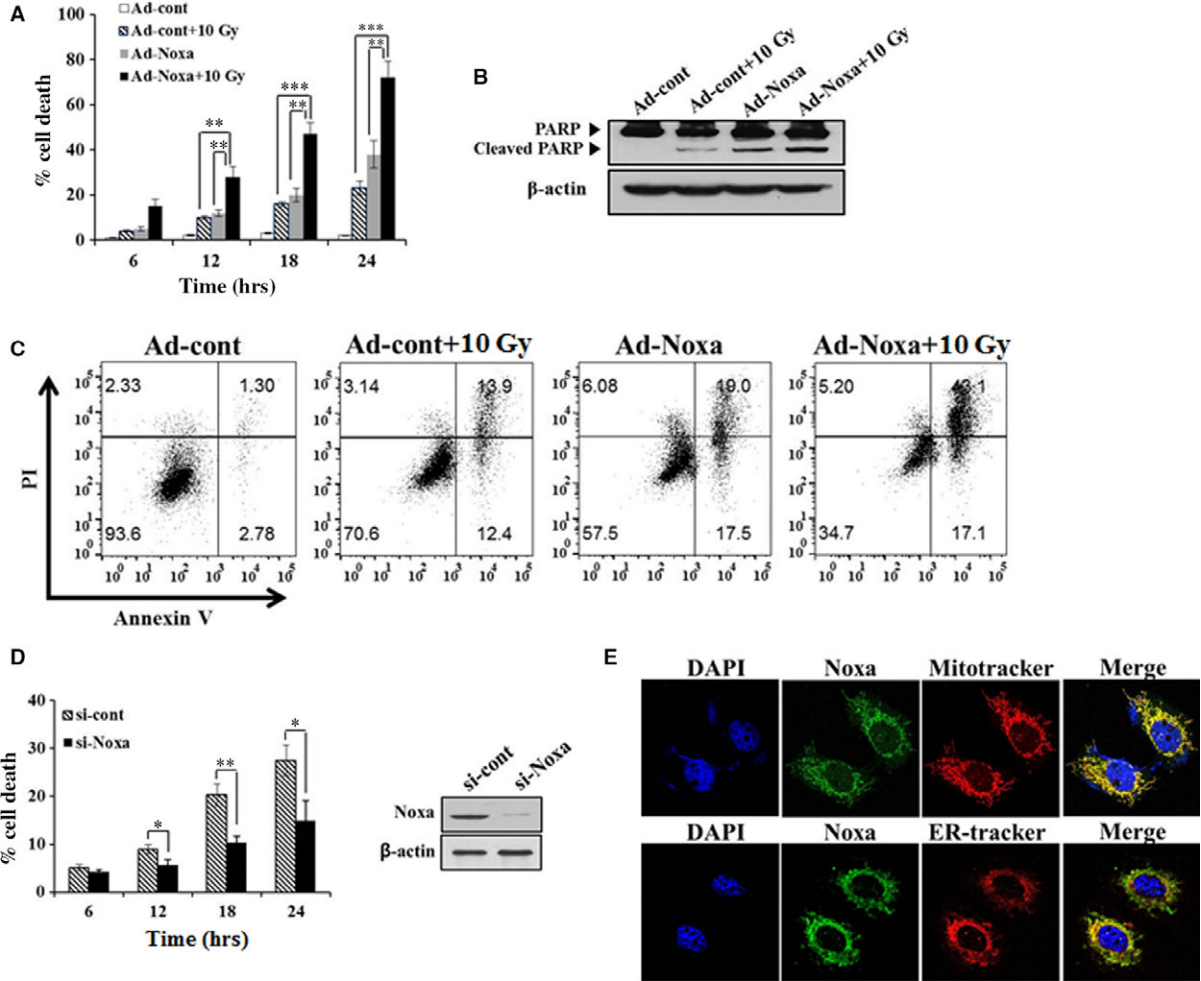
Noxa mediates cell death in response to X‐rays. (**A**) L132 human lung epithelial cells were infected with Ad‐Noxa or a control virus. After 18 hrs of infection, cells were treated with X‐rays at doses of 0 or 10 Gy at the indicated time‐points. Cell death was investigated using the WST‐1‐based cytotoxicity assay. Ad‐Noxa or control virus infected cells were treated with 10 Gy and harvested at 24 hrs for following analysis. The cell lysates were analysed using Western blot to detect total or cleaved form of PARP (**B**). The cells were double stained with Annexin V‐FITC/PI and then analysed by flow cytometry (**C**). (**D**) Cells were treated with Noxa siRNA for 48 hrs, and treated with X‐rays at doses of 0 or 10 Gy at the indicated time‐points. Cell death was investigated using the WST‐1‐based cytotoxicity assay (left). Representative western blotting of Noxa expression after transfection with siRNA (right). *P < 0.05, **P < 0.01, ***P < 0.001. (**E**) After 24 hrs of 10 Gy irradiation, L132 cells were double stained with anti‐Noxa antibody (green) and Mito Tracker (Red) or ER‐tracker (Red). The images were merged to investigate of the subcellular localization of Noxa.

Alveolar epithelial cell death has been recognized in the lungs of animals with lung injury and subsequent fibrosis 10, 16. To investigate the role of Noxa in AEC death after X‐ray irradiation, we performed an overexpression and suppression assay in L132 human lung epithelial cells. L132 cells were infected with Ad‐Noxa or Ad‐cont virus for 18 hrs and then the cells were exposed to X‐rays at a dose of 10 Gy for 6, 12, 18 and 24 hrs. Cell death rates were determined using the WST‐1 assay. As presented in Figure 4A, the Ad‐Noxa‐infected cells that had been exposed to X‐rays (10‐Gy dose) showed significantly increased cell death rates in comparison with the Ad‐cont‐infected cells that had been exposed to X‐rays (10‐Gy dose) or the Ad‐Noxa infected cells that had not. To investigate the role of Noxa in radiation‐induced apoptosis, we performed western blot for PARP cleavage and Annexin V/PI double staining. Annexin V^+^/PI^−^ and Annexin V^+^/PI^+^ proportion indicated early and late apoptotic cells respectively. As shown in Figure 4B and C, Noxa overexpression induced more apoptosis in X‐ray‐irradiated human and mouse (Fig. S1) cells, suggesting that Noxa facilitates apoptosis following radiation injury. Next, we examined knockdown of Noxa expression with siRNA (si‐Noxa) might decrease cell death after X‐ray irradiation. Knockdown of Noxa expression protected the cells from X‐ray injury (Fig. 4D). Taken together, our results indicate Noxa mediated X‐ray‐induced apoptosis. It has been reported that Noxa localizes to both mitochondria and endoplasmic reticulum (ER) in association with the induction of mitochondrial‐ and non‐mitochondrial‐dependent apopotosis 17, 18. To confirm the subcellular localization of Noxa, we performed immunofluorescence staining with anti‐Noxa antibody following the staining of mitochondria or ER with Mito Tracker or ER‐tracker respectively. As shown in Figure 4E, Noxa completely colocalized to the mitochondria with a punctate staining pattern, suggesting that Noxa are involved in mitochondrial‐dependent X‐ray induced apoptosis. In addition, Noxa partially localized to the ER, suggesting that they are involved in the induction of ER stress. To investigate the role of Noxa in ER stress, L132 cells were infected with Ad‐Noxa or transfected with si‐Noxa and then treated with X‐rays at a dose of 10 Gy for 24 hrs. The cells were then analysed using western blot with anti‐caspase 5 antibodies. Ad‐Noxa cells induced the cleavage of caspase 5, whereas si‐Noxa cells suppressed cleavage of caspase‐5 (Fig. S2), suggesting Noxa‐induced apoptosis was associated with ER stress.

### Noxa mediates cell death by ROS generation

**Figure 5:**
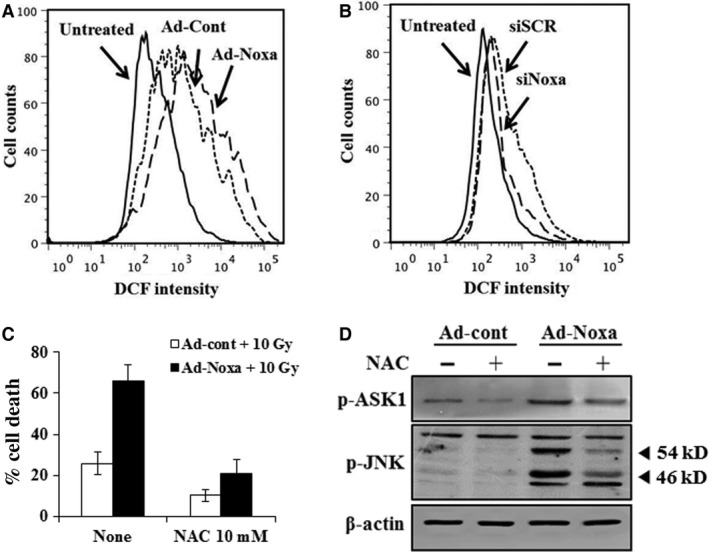
Reactive oxygen species (ROS) mediate Noxa‐induced cell death. L132 cells were treated with X‐rays at doses of 0 or 10 Gy for 6 hrs after infection with Ad‐Noxa (**A**) or transfection with Noxa siRNA (**B**). Cells were stained with 2ʹ,7ʹ‐dichlorofluorescein diacetate and subjected to flow cytometric analysis. L132 cells were infected with Ad‐Noxa for 18 hrs, and then treated with or without *N*‐acetylcysteine for an additional 3 hrs. Cells were treated with X‐rays at doses of 10 Gy for 24 hrs, and then cell death was investigated using the WST‐1‐based cytotoxicity assay (**C**) and analysed by western blot using anti‐phospho‐ASK1 (Thr845) and phosphor‐JNK antibodies (**D**).

Reactive oxygen species plays a central role in cell injuries in response to X‐rays leading to progressive fibrosis 19. In addition, it has been reported that Noxa mediates cell death through ROS generation by hypoxic injury 20. Therefore, we infected L132 cells with Ad‐Noxa and the ROS level was determined using 2ʹ,7ʹ‐dichlorofluorescein diacetate by flow cytometry analysis. As shown in Figure 5A, the ROS level was increased in Ad‐Noxa‐infected cells compared with Ad‐cont‐infected cells. The si‐Noxa cells suppressed ROS levels, whereas the si‐cont cells failed to reduce ROS levels (Fig. 5B). To investigate that ROS contributed to Noxa‐mediated X‐ray‐induced cell death, cells were infected with Ad‐Noxa with or without antioxidant *N*‐acetylcysteine (NAC). *N*‐acetylcysteine protected the Ad‐Noxa‐infected cells from cell death (Fig. 5C). Our results showed that ROS is crucial in Noxa‐mediated, X‐ray‐induced cell death. Reactive oxygen species has been known to activate signalling pathways like apoptosis signal regulating kinase 1 and its downstream mitogen‐activated protein kinase pathways 21. To investigate whether Noxa‐mediated ROS might induce the activation of ASK1 and its downstream JNK, L132 cells were infected with Ad‐Noxa for 18 hrs, and then treated with or without NAC for an additional 3 hrs. Cells were treated with X‐rays at doses of 10 Gy for 24 hrs and then harvested and analysed using western blot with anti‐phospho‐ASK1 (Thr 845) and phospho‐JNK antibodies. As shown in Figure 5D, activation of ASK1 and JNK was increased in Noxa‐overexpressed cells, which was inhibited in NAC‐treated cells, suggesting Noxa‐induced ROS was involved in the activation of ASK1 and its downstream pathway.

### X‐ray‐induced fibrosis is attenuated in Noxa knockdown cells

**Figure 6:**
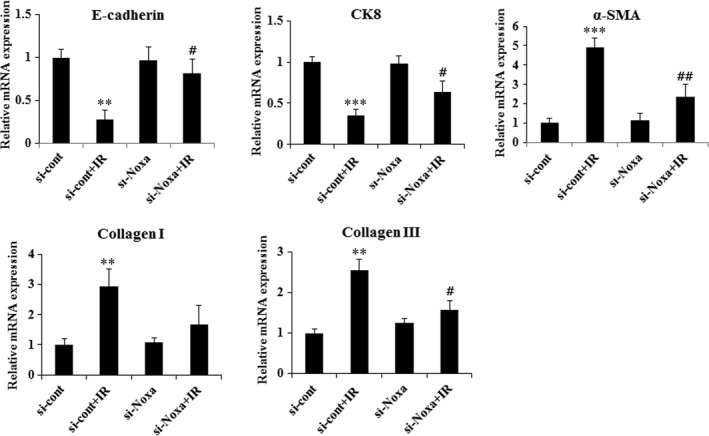
Knockdown of Noxa expression suppressed X‐ray‐induced fibrogenic markers. The si‐Noxa and si‐cont L132 cells were treated with X‐rays at a dose of 10 Gy for 48 hrs. mRNA was extracted from those cells, and RT‐PCR was performed for the indicated fibrogenic markers. **P < 0.01, ***P < 0.001 *versus* control; #*P* < 0.05, ##*P* < 0.01 *versus* control+irradiation (IR).

X‐ray irradiation obviously increases the fibrosis in the X‐ray‐irradiated lung tissues (Fig. 1C). To further assess the effects of Noxa on radiation‐induced fibrosis, we measured the expression level of fibrogenic markers in irradiated si‐Noxa‐ or si‐cont‐infected L132 cells. Cells were irradiated with X‐rays for 48 hrs, and RT‐PCR was carried out. The expression of E‐cadherin and CK8 decreased following IR, while levels of alpha‐smooth muscle actin (α‐SMA) and collagens I and III increased in the si‐cont cells. Importantly, the si‐Noxa cells showed a reduction in IR‐induced up‐regulation of α‐SMA and collagens I and III, while up‐regulation of E‐cadherin and CK8 was observed (Fig. 6A and B). These results suggest that suppression of Noxa expression attenuates fibrosis mediated by X‐ray irradiation.

### Enhanced Noxa expression in the high‐dose irradiated lung tissue

**Figure 7:**
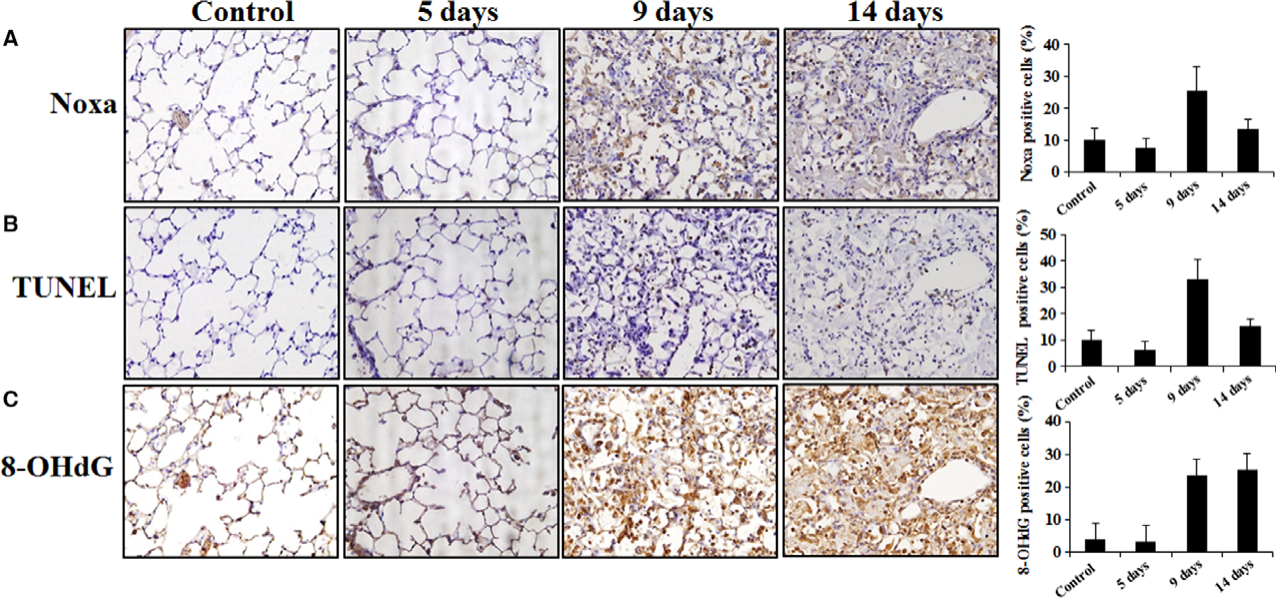
Immunohistochemistry images of Noxa from TUNEL and 8‐OHdG staining in the X‐ray‐induced mouse lung fibrosis model. Mice were killed at days 0, 5, 9 and 14 after irradiating with X‐rays at a dose of 90 Gy. Sections from irradiated lungs were immunostained with anti‐Noxa antibody (**A**). The terminal deoxynucleotidyl transferase‐mediated deoxyuridine‐5‐triphosphate‐biotin nick end labelling (TUNEL) assay was performed (**B**) and oxidative stress for 8‐OHdG (**C**) was evaluated in irradiated lung slices adjacent to those used for the analysis of Noxa expression.

We conducted immunohistochemistry to determine whether Noxa protein expression was enhanced in X‐ray‐irradiated mouse lung tissue. Enhanced Noxa protein expression was detected about 9 days after irradiation (Fig. 7A). Oxidative stress is a major driving mechanism of lung tissue damage after radiation 19. Therefore, we evaluated oxidative stress using 8‐OHdG and TUNEL assays in irradiated lung slices adjacent to those used for the analysis of Noxa expression. As shown in Figure 7B and C, X‐rays caused a significant increase in apoptotic nuclei and oxidative stress in the lung tissue cells in which Noxa expression was increased, suggesting that the degree of Noxa expression is correlated with oxidative stress and apoptosis in X‐ray‐irradiated lung tissue.

## Discussion

Recently, SBRT has been used widely for treating cancer because it delivers higher doses of radiation with improved accuracy compared with CFRT. However, radiation‐induced fibrosis is a major complication at higher radiation doses 22. This highlights the need for appropriate *in vivo* models for the study of ablative dose per fraction irradiation similar to those used with SBRT at volumes analogous to those used in human beings. In a previous study, we established an image‐guided, highly focused, small animal micro‐irradiation system that mimics clinical SBRT 14, 15. Consistent with our previous results 15, in this study, we found that high‐dose radiation exposure of the left lung caused obvious histological changes and fibrosis such as collagen deposition confined to the left lung. To gain insight into the pathogenesis of fibrosis occurring at sites of X‐ray‐induced tissue injury, we tested the hypothesis that apoptosis mediated by the BH3‐only pro‐apoptotic protein Noxa plays an essential role in X‐ray‐induced fibrosis. Apoptosis and BH3‐only pro‐apoptotic proteins have been implicated in the pathogenesis of pulmonary fibrosis 23, 24. Other groups have demonstrated that apoptosis plays an essential role for TGF‐β1‐induced fibrosis and alveolar remodelling 8, and pulmonary fibrosis animal model and IPF patients show significant lung epithelial cell injury and apoptosis 25. However, the mechanisms underlying apoptosis and fibrosis after irradiation are not well defined. In this study, we showed a new molecular mechanism underlying X‐ray‐induced fibrosis by demonstrating that the pro‐apoptotic protein Noxa‐ apoptosis plays an important role in fibrosis. Fibrogenic environmental toxins can induce apoptotic AEC death indicating that the induction of AEC apoptosis is sufficient for pulmonary fibrosis 10, and inhibition of AEC apoptosis attenuates fibrosis 7. Following irradiation, we observed that the apoptosis was increased in AECs expressing Noxa (Figs 4 and 7). Radiation induces its major biological effects through DNA damage 26. When DNA damage occurs, H2AX is quickly phosphorylated at serine 139 to generate γ‐H2AX, which aggregates at DNA damage sites forming nuclear foci. Thus, γ‐H2AX staining is a relevant analysis to measure DNA damage to provide initial evidence for cell death 27. To investigate the role of Noxa in X‐ray induced DNA damage, we performed γ‐H2AX immunofluorescence staining. Following X‐ray irradiation, γ‐H2AX level increased more in Ad‐Noxa cells compared to Ad‐Cont cells, suggesting that Noxa is involved in X‐ray‐induced DNA damage (Fig. S3). To further assess the effects of Noxa on radiation‐induced fibrosis, we measured the expression levels of collagens I and III in irradiated si‐Noxa‐ or si‐cont‐infected cells. Compared with the si‐cont cells, X‐ray irradiation decreased the expression of collagens I and III in the si‐Noxa cells. Recent studies suggest that epithelial cells undergo epithelial‐mesenchymal transition 28. Epithelial‐mesenchymal transition is characterized by increased expression of α‐SMA (the mesenchymal marker) and decreased expression of E‐cadherin and CK8 (the epithelial markers) 29. As shown in Figure 6, a decrease in α‐SAM and an increase in E‐cadherin and CK8 occurred in the si‐Noxa cells. Our results showed that radiation‐induced apoptosis and fibrosis was attenuated in Noxa knockdown cells, suggesting that Noxa‐dependent apoptosis contributes to radiation‐induced fibrosis.

Reactive oxygen species play a critical role in animal models of lung injury and fibrosis 30. Previous studies have shown that Noxa facilitates cell death through a ROS‐dependent pathway under hypoxic stress 20, indicating that ROS play a critical role in Noxa‐mediated cell death. Therefore, we have been suggested that Noxa‐induced generation of ROS might contribute to apoptosis in the irradiated cells. We found that Noxa‐induced cell death following exposure to X‐rays is mediated through ROS, because the antioxidant NAC inhibited X‐ray‐induced cell death in the Noxa‐infected cells (Fig. 5C). Although it is not clear what mechanisms are involved in the Noxa‐induced cell death pathway following irradiated to X‐rays, our findings provide clear evidence that ROS is crucial to the process. In addition, inhibition of Noxa expression prevents the production of fibrogenic markers (Fig. 6), and the TUNEL assay revealed that Noxa expression is correlated with apoptosis and oxidative stress in irradiated lung tissue (Fig. 7). These results strongly indicate that Noxa plays a role in radiation‐induced fibrosis and that ROS is involved in this process. In summary, the present studies indicate that our image‐guided small animal micro‐irradiation system that mimics clinical SBRT represents an appropriate model for investigating radiation‐induced lung injury. The significant increasing in the levels of Noxa appears to be associated with the radiation‐induced lung injury in mice. Inhibition of Noxa expression protected cells from apoptosis, and improved resistance to the radiation‐induced increase in ROS and the production of fibrogenic markers. This suggests that following radiotherapy, Noxa expression in patients may serve as an important indicator of the risk of radiation‐induced lung damage. Our results suggest a previously unknown link between the apoptosis of AECs through Noxa and the fibrosis response elicited by exposure to radiation. Further research is required into the specific mechanisms involved in radiation‐induced, Noxa‐mediated fibrosis using mice that are deficient in Noxa.

## Conflict of interest

The authors declare no conflict of interest.

## Supporting information


**Figure S1** Noxa mediates cell death in response to X‐rays.

**Figure S2** Noxa‐induced cell death is associated with ER stress.

**Figure S3** Noxa facilitates DNA damage in response to X‐rays.

**Table S1** Microarray results for pro‐apoptotic genes.

## References

[1] Koto M , Takai Y , Ogawa Y , *et al* A phase II study on stereotactic body radiotherapy for stage I non‐small cell lung cancer. Radiother Oncol. 2007; 85: 429–34. 18022720 10.1016/j.radonc.2007.10.017

[2] Timmerman R , Abdulrahman R , Kavanagh BD , *et al* Lung cancer: a model for implementing stereotactic body radiation therapy into practice. Front Radiat Ther Oncol. 2007; 40: 368–85. 17641520 10.1159/000106047

[3] Pfister DG , Johnson DH , Azzoli CG , *et al* American Society of Clinical Oncology treatment of unresectable non‐small‐cell lung cancer guideline: update 2003. J Clin Oncol. 2004; 22: 330–53. 14691125 10.1200/JCO.2004.09.053

[4] Hong ZY , Eun SH , Park K , *et al* Development of a small animal model to simulate clinical stereotactic body radiotherapy‐induced central and peripheral lung injuries. J Radiat Res. 2014; 55: 648–57. 24556815 10.1093/jrr/rrt234 PMC4099992

[5] Elias JA , Zhu Z , Chupp G , *et al* Airway remodeling in asthma. J Clin Invest. 1999; 104: 1001–6. 10525034 10.1172/JCI8124 PMC408860

[6] Kuwano K , Hagimoto N , Kawasaki M , *et al* Essential roles of the Fas‐Fas ligand pathway in the development of pulmonary fibrosis. J Clin Invest. 1999; 104: 13–9. 10393694 10.1172/JCI5628 PMC408400

[7] Budinger GR , Mutlu GM , Eisenbart J , *et al* Proapoptotic Bid is required for pulmonary fibrosis. Proc Natl Acad Sci USA. 2006; 103: 4604–9. 16537427 10.1073/pnas.0507604103 PMC1401229

[8] Lee CG , Cho SJ , Kang MJ , *et al* Early growth response gene 1‐mediated apoptosis is essential for transforming growth factor beta1‐induced pulmonary fibrosis. J Exp Med. 2004; 200: 377–89. 15289506 10.1084/jem.20040104 PMC2211975

[9] Osterholzer JJ , Olszewski MA , Murdock BJ , *et al* Implicating exudate macrophages and Ly‐6C(high) monocytes in CCR2‐dependent lung fibrosis following gene‐targeted alveolar injury. J Immunol. 2013; 190: 3447–57. 23467934 10.4049/jimmunol.1200604 PMC3608799

[10] Sisson TH , Mendez M , Choi K , *et al* Targeted injury of type II alveolar epithelial cells induces pulmonary fibrosis. Am J Respir Crit Care Med. 2010; 181: 254–63. 19850947 10.1164/rccm.200810-1615OC PMC2817814

[11] Zhang Y , Zhang X , Rabbani ZN , *et al* Oxidative stress mediates radiation lung injury by inducing apoptosis. Int J Radiat Oncol Biol Phys. 2012; 83: 740–8. 22270165 10.1016/j.ijrobp.2011.08.005 PMC3649017

[12] Shivshankar P , Brampton C , Miyasato S , *et al* Caveolin‐1 deficiency protects from pulmonary fibrosis by modulating epithelial cell senescence in mice. Am J Respir Cell Mol Biol. 2012; 47: 28–36. 22362388 10.1165/rcmb.2011-0349OC PMC3402795

[13] Oda E , Ohki R , Murasawa H , *et al* Noxa, a BH3‐only member of the Bcl‐2 family and candidate mediator of p53‐induced apoptosis. Science. 2000; 288: 1053–8. 10807576 10.1126/science.288.5468.1053

[14] Cho J , Kodym R , Seliounine S , *et al* High dose‐per‐fraction irradiation of limited lung volumes using an image‐guided, highly focused irradiator: simulating stereotactic body radiotherapy regimens in a small‐animal model. Int J Radiat Oncol Biol Phys. 2010; 77: 895–902. 20510200 10.1016/j.ijrobp.2009.12.074

[15] Hong ZY , Lee HJ , Choi WH , *et al* A preclinical rodent model of acute radiation‐induced lung injury after ablative focal irradiation reflecting clinical stereotactic body radiotherapy. Radiat Res. 2014; 182: 83–91. 24937781 10.1667/RR13535.1

[16] Matute‐Bello G , Liles WC , Steinberg KP , *et al* Soluble Fas ligand induces epithelial cell apoptosis in humans with acute lung injury (ARDS). J Immunol. 1999; 163: 2217–25. 10438964

[17] Li J , Lee B , Lee AS . Endoplasmic reticulum stress‐induced apoptosis: multiple pathways and activation of p53‐up‐regulated modulator of apoptosis (PUMA) and NOXA by p53. J Biol Chem. 2006; 281: 7260–70. 16407291 10.1074/jbc.M509868200

[18] Hassan M , Alaoui A , Feyen O , *et al* The BH3‐only member Noxa causes apoptosis in melanoma cells by multiple pathways. Oncogene. 2008; 27: 4557–68. 18408751 10.1038/onc.2008.90

[19] Fleckenstein K , Zgonjanin L , Chen L , *et al* Temporal onset of hypoxia and oxidative stress after pulmonary irradiation. Int J Radiat Oncol Biol Phys. 2007; 68: 196–204. 17448873 10.1016/j.ijrobp.2006.12.056 PMC1939695

[20] Kim JY , Ahn HJ , Ryu JH , *et al* BH3‐only protein Noxa is a mediator of hypoxic cell death induced by hypoxia‐inducible factor 1alpha. J Exp Med. 2004; 199: 113–24. 14699081 10.1084/jem.20030613 PMC1887730

[21] Ichijo H , Nishida E , Irie K , *et al* Induction of apoptosis by ASK1, a mammalian MAPKKK that activates SAPK/JNK and p38 signaling pathways. Science. 1997; 275: 90–4. 8974401 10.1126/science.275.5296.90

[22] Fuks Z , Kolesnick R . Engaging the vascular component of the tumor response. Cancer Cell. 2005; 8: 89–91. 16098459 10.1016/j.ccr.2005.07.014

[23] Kang HR , Cho SJ , Lee CG , *et al* Transforming growth factor (TGF)‐beta1 stimulates pulmonary fibrosis and inflammation *via* a Bax‐dependent, bid‐activated pathway that involves matrix metalloproteinase‐12. J Biol Chem. 2007; 282: 7723–32. 17209037 10.1074/jbc.M610764200

[24] Calabrese F , Giacometti C , Beghe B , *et al* Marked alveolar apoptosis/proliferation imbalance in end‐stage emphysema. Respir Res. 2005; 6: 14. 15705190 10.1186/1465-9921-6-14 PMC549521

[25] Cheresh P , Kim SJ , Tulasiram S , *et al* Oxidative stress and pulmonary fibrosis. Biochim Biophys Acta. 2013; 1832: 1028–40. 23219955 10.1016/j.bbadis.2012.11.021 PMC3639303

[26] Lomax ME , Folkes LK , O'Neill P . Biological consequences of radiation‐induced DNA damage: relevance to radiotherapy. Clin Oncol (R Coll Radiol). 2013; 25: 578–85. 23849504 10.1016/j.clon.2013.06.007

[27] Plesca D , Mazumder S , Almasan A . DNA damage response and apoptosis. Methods Enzymol. 2008; 446: 107–22. 18603118 10.1016/S0076-6879(08)01606-6 PMC2911482

[28] Radisky DC . Epithelial‐mesenchymal transition. J Cell Sci. 2005; 118: 4325–6. 16179603 10.1242/jcs.02552

[29] Willis BC , duBois RM , Borok Z . Epithelial origin of myofibroblasts during fibrosis in the lung. Proc Am Thorac Soc. 2006; 3: 377–82. 16738204 10.1513/pats.200601-004TK PMC2658689

[30] Zhao W , Diz DI , Robbins ME . Oxidative damage pathways in relation to normal tissue injury. Br J Radiol. 2007; 80: S23–31. 17704323 10.1259/bjr/18237646

