# Supplementary figures and images for: Pro‐apoptotic Noxa is involved in ablative focal irradiation‐induced lung injury
Source: J Cell Mol Med. 2016 Nov 15;21(4):711–9. doi: 10.1111/jcmm.13014 (PMC5345661; doi:10.1111/jcmm.13014)

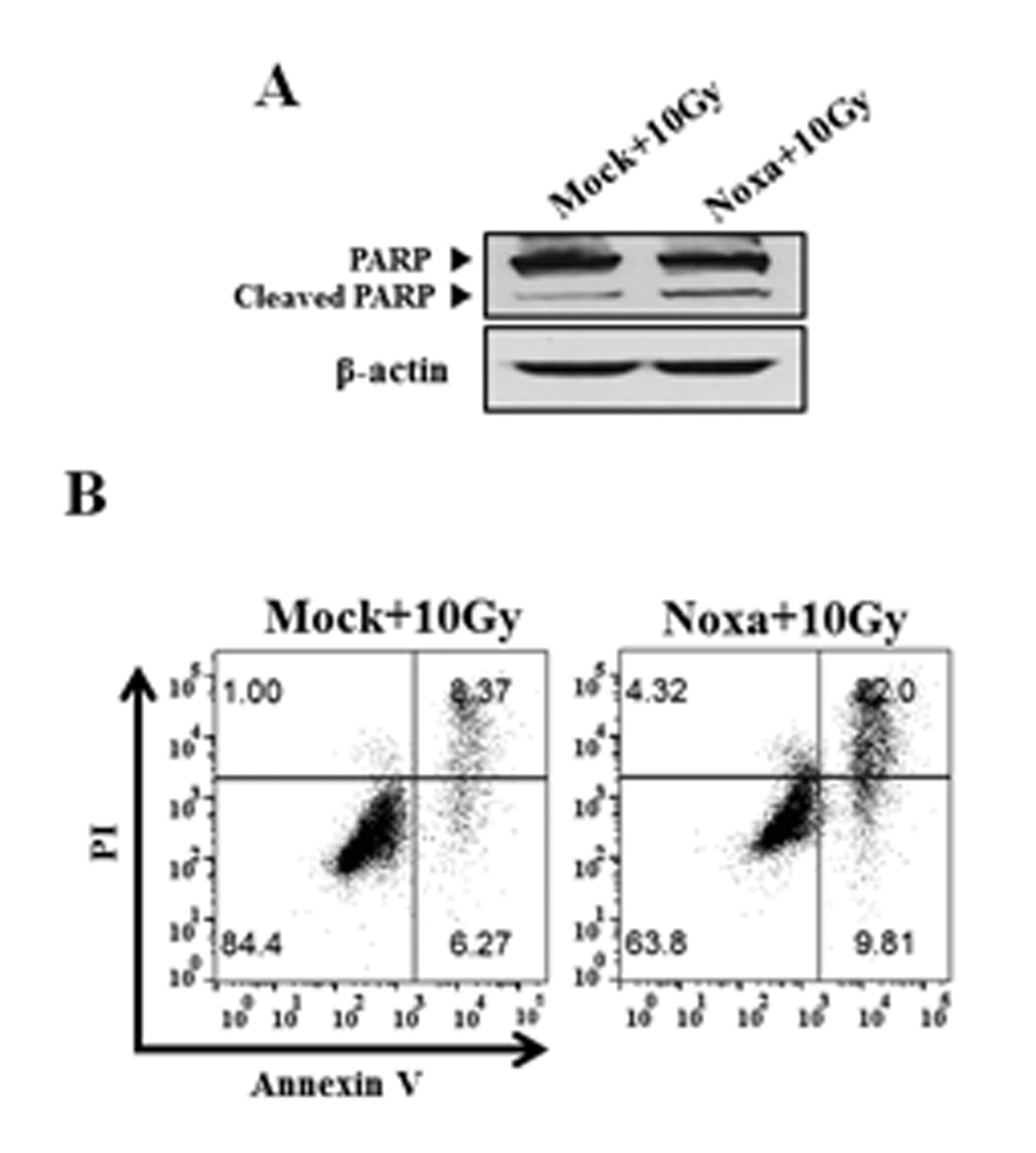

Supplement: Supplementary file 1 — Figure S1 Noxa mediates cell death in response to X‐rays. [file JCMM-21-711-s001.tif]

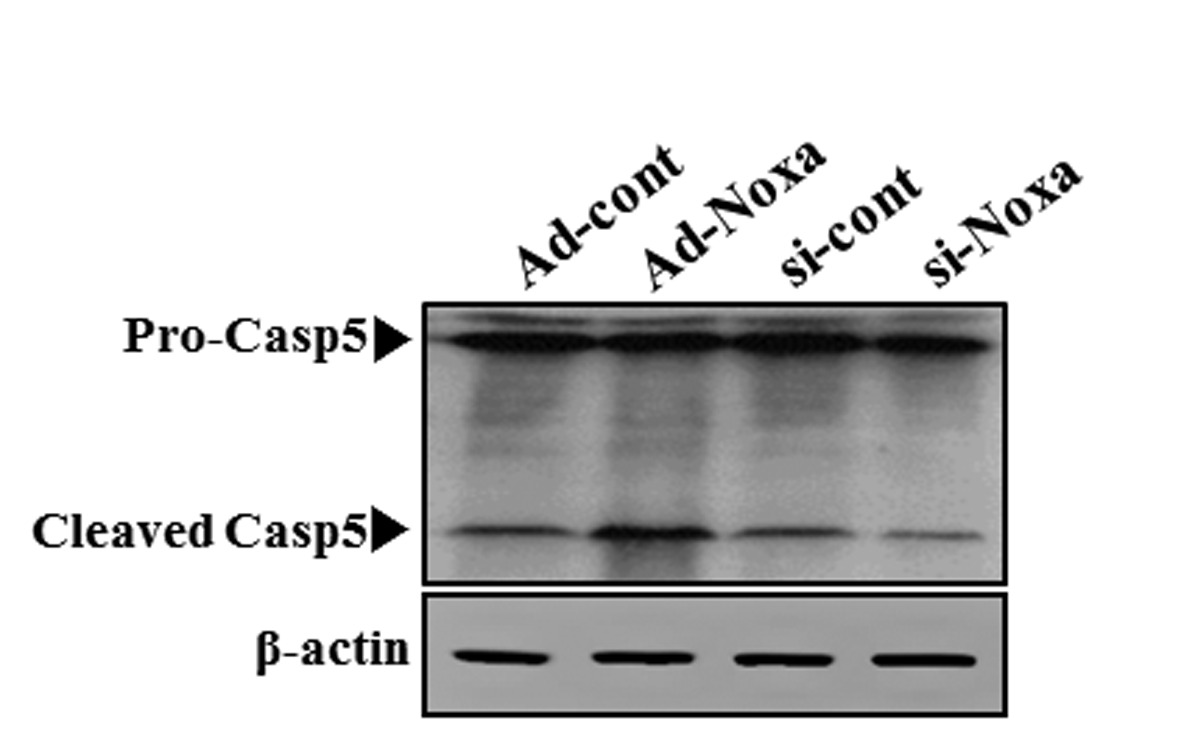

Supplement: Supplementary file 2 — Figure S2 Noxa‐induced cell death is associated with ER stress. [file JCMM-21-711-s002.tif]

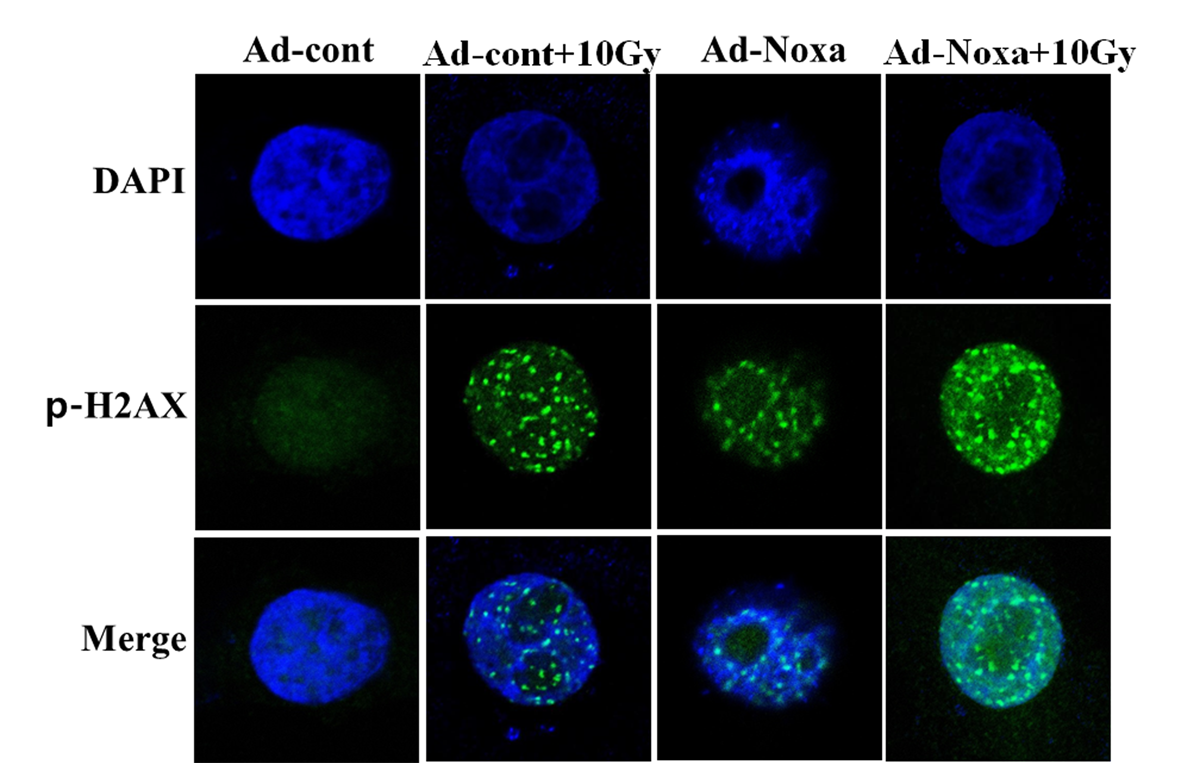

Supplement: Supplementary file 3 — Figure S3 Noxa facilitates DNA damage in response to X‐rays. [file JCMM-21-711-s003.tif]
