# Supplementary material for: Pro‐apoptotic Noxa is involved in ablative focal irradiation‐induced lung injury
Source: J Cell Mol Med. 2016 Nov 15;21(4):711–9. doi: 10.1111/jcmm.13014 (PMC5345661; doi:10.1111/jcmm.13014)
Supplement: Supplementary file 4 — Table S1 Microarray results for pro‐apoptotic genes. [file JCMM-21-711-s004.docx]

**Supplementary Table S1. Microarray results for pro-apoptotic genes**

| **Gene Name** | **Description** | **Log Fold Change** | | **RefSeq** |
| --- | --- | --- | --- | --- |
|  |  | |  |  |
| Pmaip1 | phorbol-12-myristate-13-acetate-induced protein 1 | | 3.109382 | NM_021451 |
| Bok | BCL2-related ovarian killer protein | | 1.450258 | NM_016778 |
| Bak1 | BCL2-antagonist/killer 1 | | 2.627049 | NM_007523 |
| Bad | BCL2-associated agonist of cell death | | 1.503473 | NM_007522 |
| Bid | BH3 interacting domain death agonist | | 3.050189 | NM_007544 |
| Hrk | BCL2 interacting protein (contains only BH3 domain) | | 1.322132 | NM_007545 |
| Bcl2L11 | BCL2-like 11 | | 2.899062 | NM_207680 |
| Bcl2L14 | BCL2-like 14 | | 2.709732 | NM_025778 |
| Bcl2L13 | BCL2-like 13 | | 1.100007 | NM_153516 |
| BBC3 | BCL2 binding component 3 | | 1.361250 | NM_133234 |
| Bax | BCL2-associated X protein | | 4.289938 | NM_007527 |
