# Supplementary material for: Pro‐apoptotic Noxa is involved in ablative focal irradiation‐induced lung injury
Source: J Cell Mol Med. 2016 Nov 15;21(4):711–9. doi: 10.1111/jcmm.13014 (PMC5345661; doi:10.1111/jcmm.13014)
Supplement: Supplementary file 5 [file JCMM-21-711-s005.docx]

**Supplementary Materials**

**Supplementary Figure S1. Noxa mediates cell death in response to X-rays**

MLE12 mouse lung epithelial cells were transfected with Mock or pcDNA-Noxa vectors for 18 h and then exposed to X-ray at a dose of 10 Gy for 24 h. (A) The cell lysates were analyzed using Western blot to detect total or cleaved form of PARP. (B) The cells were double stained with Annexin V-FITC/PI and then analyzed by flow cytometry. Annexin V+/PI- and Annexin V+/PI+ proportion indicated early and late apoptotic cells, respectively.

**Supplementary Figure S2. Noxa-induced cell death is associated with ER stress**

L132 hunam lung epithelial cells were infected with Ad-Noxa or transfected with si-Noxa and then exposed to X-rays at a dose of 10 Gy for 24 h. The cells were then analyzed using Western blot with anti-caspase 5 antibody.

**Supplementary Figure S3. Noxa facilitates DNA damage in response to X-rays**

L132 human lung epithelial cells were infected with Ad-Noxa or a control virus. After 18 h of infection, cells were treated with X-rays at doses of 10 Gy for 24 h, and then immunostained with anti-phospho-H2AX (S139) antibody to detect radiation-induced DNA damage.
